# Correlation of Ocular Biometric Characteristics and Central Corneal Thickness With the Severity of Myopia: A Multicentric Study From Bodhya Eye Consortium, India

**DOI:** 10.7759/cureus.100478

**Published:** 2025-12-31

**Authors:** Preeti Sharma, Shailja Tibrewal, Chintan Shah, Samir Sutar, Iva Kalita, Anupam Sahu, Pradeep Agarwal, Pradhnya Sen, Atanu Majumdar, Suma Ganesh

**Affiliations:** 1 Department of Pediatric Ophthalmology, Strabismus, and Neuro-ophthalmology, Dr Shroff's Charity Eye Hospital, New Delhi, IND; 2 Pediatric Subgroup, Bodhya Eye Consortium, New Delhi, IND; 3 Department of Pediatric Ophthalmology, Sadguru Netra Chikitsalaya, Chitrakoot, IND; 4 Department of Pediatric Ophthalmology, C. L. Gupta Eye Institute, Moradabad, IND; 5 Ophthalmology, Sitapur Eye Hospital, Sitapur, IND; 6 Department of Pediatric Ophthalmology, MGM Eye Institute, Raipur, IND; 7 Department of Biostatistics, Dr Shroff’s Charity Eye Hospital, New Delhi, IND; 8 Department of Pediatric Ophthalmology, Strabismus, and Neuro-ophthalmology, Dr Shroff’s Charity Eye Hospital, New Delhi, IND

**Keywords:** axial length, biometry, central corneal thickness, keratometry, myopia

## Abstract

Purpose: To evaluate the correlation and independent associations between ocular biometric parameters and central corneal thickness with the severity of myopia in children.

Methods: This multicentric retrospective cross-sectional study included children ≤18 years of age with simple axial myopia or compound myopic astigmatism. Ocular biometry was performed using an optical biometer (IOL master, Zeiss). The data collected was age, gender, height, weight, body mass index (BMI), cycloplegic refraction (Spherical Equivalent, SE), best corrected visual acuity (BCVA), axial length (AL), anterior chamber depth (ACD), lens thickness (LT), corneal power (K), central corneal thickness (CCT), and AL/Corneal curvature (CR) ratio. The various demographic and biometric parameters were compared between the myopia categories [low (≥−3.00 D), moderate (-3.25 to ≥-6.00D), and severe (<-6D)].

Results: A total of 1095 children (2174 eyes), 579 males and 516 females, were included. Out of 1095, 16 patients had one eye myopic while the other was emmetropic. The mean age was 11.61 ± 3.73 years. Low, moderate, and severe myopia was observed in 35.53%, 29.13%, and 35.34% of children with a mean SE of -1.93±0.71, -4.35±0.84, and -10.22±3.70, respectively. The severe myopia group showed the lowest mean values for age, height, weight, BMI, and the poorest mean BCVA (p < 0.001). Axial length demonstrated a strong association with SE, with increasing AL corresponding to higher degrees of myopia (estimate = -2.53, p < 0.001). Steeper mean keratometry (estimate = -1.01, p < 0.001), reduced ACD (estimate = 2.73, p < 0.001), and greater LT (estimate = -1.63, p = 0.001) were also significantly associated with more severe myopia. In contrast, CCT did not show an independent association with SE (p = 0.743). Among demographic variables, height showed a modest but significant relationship, with taller children associated with slightly lower degrees of myopia (estimate = 3.70, p < 0.001). Parental myopia and other covariates did not retain statistical significance after multivariable adjustment.

Conclusion: In this large multicentric cohort of Indian children, ocular biometric parameters, particularly AL, CR, ACD, and LT were independently associated with the severity of myopia. The CCT showed no significant relationship. These findings underscore the importance of comprehensive ocular biometric profiling beyond SE alone for understanding myopia severity. Population-specific biometric data may aid in improved risk stratification and individualised myopia management.

## Introduction

Myopia is the most common eye disorder and has become a significant public health concern affecting populations worldwide [1,2]. The burden is specifically high in Asia, where prevalence increases to >80% in some Eastern Asian countries [3,4]. Children and young adults are increasingly affected by this disease with a similar global distribution [3,4]. Younger onset and higher myopia grade at onset are often associated with a greater myopia progression [5].

High myopia, which is defined as an axial length (AL) ≥26 mm or spherical equivalent (SE) ≤−6.00 dioptres (D) when the accommodation is relaxed, has a prevalence of 2% to 9% in the general population [1,6]. The progression of simple myopia to high myopia converts it from a simple refractive error to a vision-threatening condition. It is associated with progressive retinal pathologic changes, presenile cataracts, and glaucoma. The prevalence of pathological myopia is also alarmingly high in the Asian region [1,7]. Various genetic and environmental factors have been implicated in these regional differences in myopia's prevalence, severity, and progression [8]. A large genome-wide association study (GWAS) has identified numerous variants across more than 400 loci associated with refractive error and myopia [9]. Several of these variants show different effect sizes or frequencies across ethnic groups, which explains the higher prevalence and more severe myopia in East Asian populations compared with Europeans. The geographic variations in myopia prevalence are also linked to education intensity, increased near-work demands, and reduced time spent outdoors, rather than purely population genetics [10,11]. Evidence suggests that genetic factors may determine an individual’s susceptibility to environmental factors, implying that modifying these key lifestyle risk factors may reduce the burden of myopia onset and progression, even in genetically susceptible populations [12-14].

The key ocular biometric determinants of refractive error include AL, corneal curvature (CR), anterior chamber depth (ACD), and the dioptric power of the lens [15]. An increase in AL with age is compensated for by decreased corneal dioptric power (K) and lens thickness (LT) [15]. The coordination of the ocular biometric parameters is partly controlled by genetic factors. Multiple genes influence eye growth and act through small, additive effects on a shared eye growth/eye size pathway [16,17]. Single-nucleotide polymorphisms in genes like GJD2, ZC3H11B, RSPO1, and WNT7B have been implicated in elongation of AL in children predisposing them to myopia [18-21]. Most of these genes demonstrate retinal expression and play key roles in cell-to-cell signaling pathways. They support the existing concept that light-driven retinal signals initiate a retina-to-sclera cascade, ultimately leading to scleral remodeling and the development of refractive errors [22].

A discordance between the changes in the biometric determinants with age leads to different refractive errors. The changes in the myopic eye are unique compared to those in non-myopic eyes [15,23-29]. Axial length has been shown to be the predominant factor determining the onset and progression of myopia, wherein a greater AL is associated with a higher myopia [15,30]. However, the axial length to corneal curvature (AL/CR) ratio may be a stronger indicator than simply AL of myopia [31]. The relationship of the CR, LT, and ACD with the refractive error is less linear than the AL [15]. Previous studies have shown conflicting associations between these ocular biometric parameters and the degree of myopia [15,28,32,33]. Considering the genetic and environmental determinants of eye growth and refractive error, ethnic variation may help explain discrepancies across different studies. Few studies have described the ocular biometric characteristics of myopic children in different ethnic populations [15,23-28,32,34]. Understanding ocular biometric parameters in the Indian myopic population is particularly important because existing myopia literature and biometric norms are largely derived from East Asian and Western cohorts, which may not accurately reflect Indian ocular anatomy [35,36]. Data from the Indian population remains limited, with no studies correlating these parameters with the severity of myopia [15,26,27,37]. Moreover, there is a paucity of large-scale multi-institutional biometric correlation data from India. We hypothesised that AL, AL/CR ratio, LT, ACD, CR, and CCT would be independently associated with the severity of myopia in Indian children. A multicentric study of the ocular biometric characteristics and their association with the degree of myopia was thus undertaken to understand the changes occurring during myopia development.

## Materials and methods

This was a multicenter, retrospective study involving the following five institutions: Dr Shroff’s Charity Eye Hospital, Delhi, Sadguru Netra Chikitsalaya, Madhya Pradesh, C. L. Gupta Eye Institute, Uttar Pradesh, Sitapur Eye Hospital, Uttar Pradesh, and MGM Eye Institute, Chhattisgarh. These institutes are part of the Bodhya Eye Consortium, which is a group of research-oriented tertiary eye care institutes in India. The study was approved by the Institutional Review Board of Dr Shroff’s Charity Eye Hospital, New Delhi (IRB/2022/SEP/129), and was conducted in accordance with the tenets of the Helsinki Declaration. Consensus-led proformas were prepared and used for data collection across all the participating institutes. Patient data was anonymised before sharing and subsequent analysis.

All children (0-18 years) with myopia who underwent ocular biometry between January 2020 and December 2022 were included in the study. Myopia was defined as a SE of ≤-0.5D. Patients with simple myopic astigmatism, corneal scar, previous intraocular surgery, ectopia lentis, microspherophakia, and retinal detachment were excluded. Amblyopia and strabismus were not excluded. The age, gender, height, weight, parental myopia, h/o prematurity, h/o hours of near activities and outdoor activities, type and magnitude of myopia, and best corrected visual acuity (BCVA) were noted from the patient’s medical records. Prematurity was defined as gestational age less than 37 weeks [38]. Ocular biometric parameters recorded were AL, ACD, LT, corneal dioptric power (K), and central corneal thickness (CCT). 

Ocular biometry was performed using optical low-coherence reflectometry (IOL Master, Carl Zeiss Meditec AG, Jen, Germany) at all the participating centres. The IOL master 700 was used in four centres, while the IOL master 500 was used in one centre. Both machines provide an average of five measurements with a traffic-light system (green, yellow, red) to indicate the quality of the scans. At all the participating centres, trained optometrists performed the measurements. A signal-to-noise ratio of greater than 2.1 and a standard deviation of less than 0.027 for AL was considered a good scan. Additionally, the IOL master 700 allows for visualisation of the fovea to confirm alignment with the patient’s visual axis. Cycloplegic retinoscopy performed within six weeks of the date of biometry was considered. Cycloplegia was achieved by instillation of cyclopentolate 1% eye drop two times spaced 15 minutes apart in children below 14 years of age and tropicamide 1% in those older than 14 years of age. The SE was calculated using the formula SE = sphere + ½ cylinder. Myopia was graded into low (≥−3.00 D), moderate (-3.25 to -6.00D), and severe (<-6D). The average K was calculated as the mean of the flattest (K1) and steepest (K2) corneal dioptric power. The corneal radius of curvature (CR) was calculated using the formula CR in mm = 337.5/corneal dioptric power (in Dioptres). The AL/CR ratio was calculated by dividing the AL by the mean CR. The body mass index (BMI) was calculated using the formula BMI = Weight (kg) / Height (m)²

Statistical methods

The sample size for the study was determined to estimate a correlation coefficient with a desired confidence interval. It was based on the distribution of Z, where Z is Fisher’s Z transformation of the sample correlation coefficient, r. We used the most conservative 95% confidence interval for the sample mean of Z corresponding to a 95% confidence interval of ±0.10 for the sample correlation coefficient. A design effect of 2 was assumed, giving a minimum statistical sample size of 800. However, we planned to include as many cases as possible beyond this minimum, within the time period allocated for the study.

Continuous variables were summarised using means and standard deviations, and categorical variables using frequencies and percentages. The Kruskal-Wallis test was used to assess differences in ocular biometry across age-gender groups and across the severity levels of myopia (mild, moderate, and severe). The strength of the association between optical biometry and SE was measured using Pearson’s correlation as a part of the univariate analysis. The strength of association between optical biometry parameters and spherical equivalent was also examined using multivariable linear regression. Spherical equivalent values were recorded with their original negative signs. Initially, all physical characteristics and optical biometry parameters were included as covariates, and stepwise backward elimination was used to remove variables that did not contribute meaningfully. Highly collinear variables were excluded based on their VIF values. A p-value of <0.05 was considered statistically significant. All analyses were performed using R software version 4.3.2 (R Foundation for Statistical Computing, Vienna, Austria).

## Results

**Figure 1:**
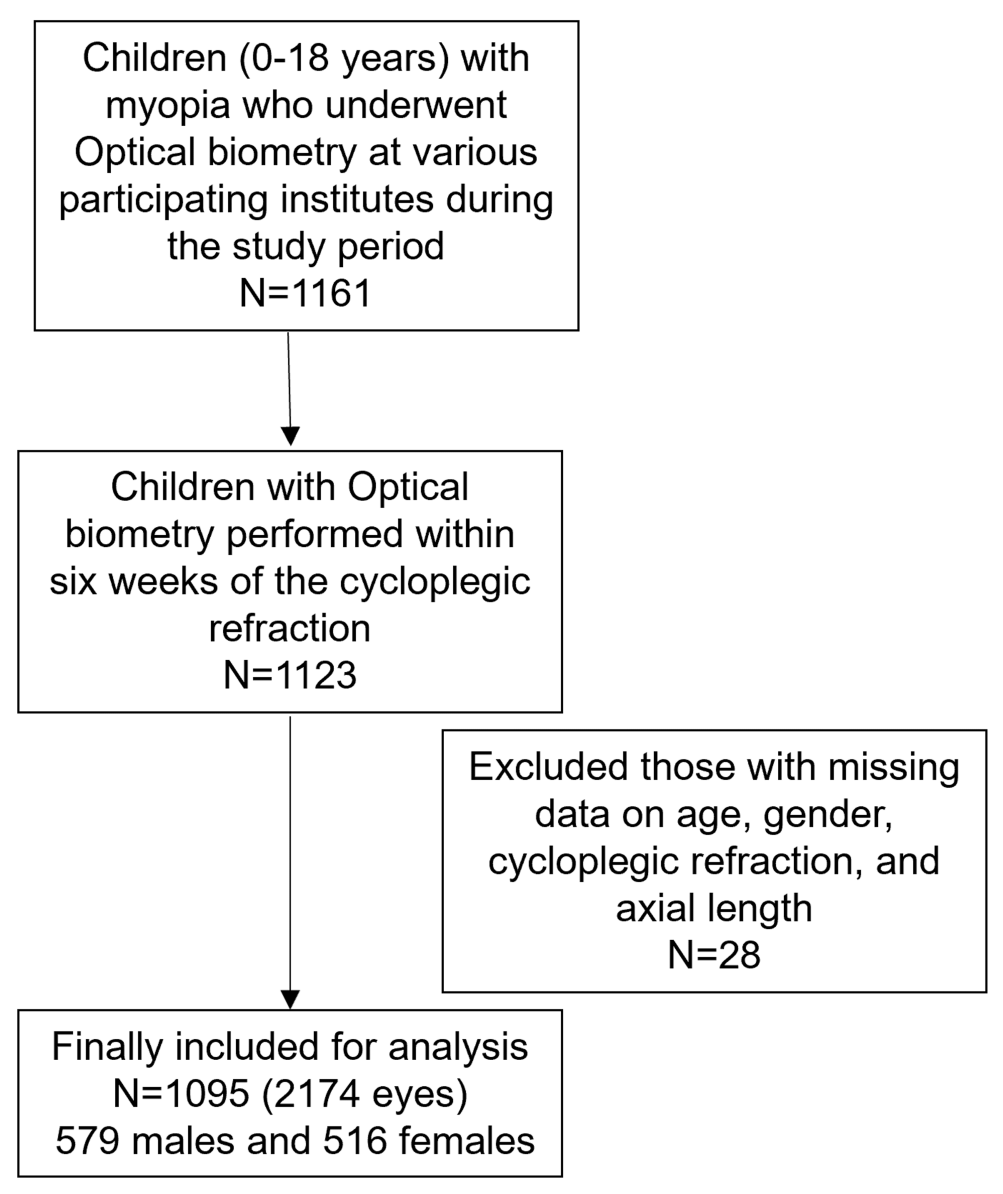
Flowchart of the patients included in the study after application of the eligibility criteria

A total of 1095 children, 516 females (47.12%) and 579 males (52.88%) were included. Figure 1 shows the flow diagram of the patients included in the study.

The mean age of the patients was 11.61 ± 3.73 years (three to 18 years). There were 75 children (7%) in the three to five years age group, 318 children (29%) in the five to 10 years age group, 528 children (48%) in the 10 to 15 years age group, and 174 children (16%) in the 15 to 18 years age group. The data regarding parental myopia was available for 784 patients. At least one parent was myopic in 27% (212) of the patients, both parents in 8.04%, only the father in 8.16%, and only the mother in 10.84% of patients. Data regarding prematurity was available in 715 records. Preterm birth was noted in 0.98% of the patients. Table 1 shows the detailed demographic characteristics of the children who participated in the study, along with sample proportions and absolute numbers. 

**Table 1:** Demographic and clinical characteristics of the patients included in the study

Parameter	Mean ± Standard Deviation	N
Age (years)	11.61 ± 3.73	1095
Height (meters)	1.37 ± 0.23	537
Weight (kg)	36.84 ± 14.46	666
Outdoor Activities (hours)	1.42 ± 1.76	668
Near Activities (hours)	6.05 ± 4.15	604
Gender	Frequency	Percentage
Female	516	47.12
Male	579	52.88
Total	1095	100
Parental Myopia	Frequency	Percentage
Both	63	8.04
Father Only	64	8.16
Mother Only	85	10.84
At least one	212	27.04
None	572	72.96
Total	784	100
Gestation	Frequency	Percentage
Pre-term	7	0.98
Complete	708	99.02
Total	715	100
Worse Eye Myopia	Frequency	Percentage
Mild	389	35.53%
Moderate	319	29.13%
Severe	387	35.34%
Total Cases	1095	100.00%
Bilateral condition	Frequency	Percentage
Normal + Mild	11	1.00%
Normal + Moderate	5	0.46%
Normal + Severe	8	0.73%
Mild + Mild	378	34.52%
Mild + Moderate	75	6.85%
Mild + Severe	20	1.83%
Moderate + Moderate	239	21.83%
Moderate + Severe	75	6.85%
Severe + Severe	284	25.94%
Total Cases	1095	100.00%

**Figure 2:**
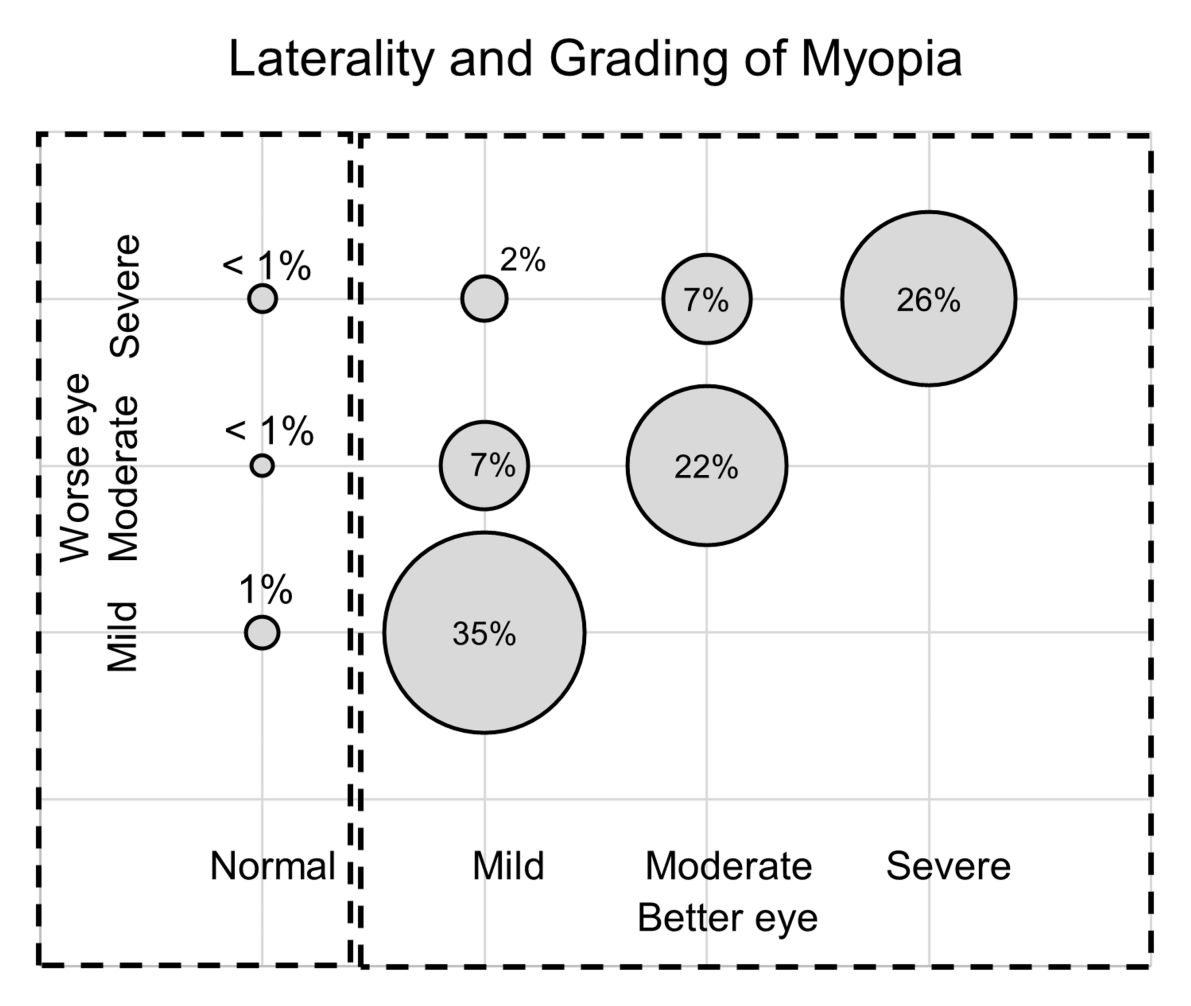
The distribution of the grade of myopia between the two eyes of the 1095 children included in the study

Out of the 1095 children, 389 (35.53%) had mild myopia, 319 (29.13%) had moderate myopia, and 387 (35.34%) had severe myopia when classified by their worst eye condition, respectively. Over 35% of the children had severe myopia in at least one eye, and 26% had severe myopia in both eyes. The majority (98%) of the children had bilateral myopia. A similar grade of myopia in both eyes was observed in 82.28% of patients (Figure 2), while 15.72% had different levels of severity in each eye. Sixteen of the total 2,190 eyes were emmetropic. Out of the 2,174 myopic eyes, 40% were mildly myopic, 29% were moderately myopic, and 31% were severely myopic. The average myopia was -5.19±4.11D in total eyes, -1.93±0.71D in the mild myopia group, -4.35±0.84D in the moderate myopia group, and -10.22±3.70D in the severe myopia group respectively.

Table 2 shows the vision, refractive error, and ocular biometry across different age-sex categories of the children included in the study. The proportions of children with mild, moderate, and severe myopia, along with all vision and ocular biometry parameters (except for the lens thickness), varied significantly across age-sex categories. The highest proportions of children with severe myopia were found in the youngest age group, with 59% among both boys and girls aged three to five years. These children had the worst BCVA (average logMAR 0.37 ± 0.31 for boys and 0.38 ± 0.29 for girls) and the highest degree of myopia (SE of -8.09 ± 5.22 for boys and -8.12 ± 5.04 for girls). This group also recorded the longest AL and the highest AL/CR ratio (25.49 ± 1.97 and 3.34 ± 0.24, respectively, for boys and 24.84 ± 1.93 and 3.32 ± 0.26, respectively, for girls).

**Table 2:** Refractive error, vision, and ocular biometry across various age-sex categories CI: Confidence Interval, BCVA: Best Corrected Visual Acuity, SD: Standard Deviation, SE: Spherical Equivalent, AL/CR ratio: Axial Length to Corneal Curvature ratio, K: Keratometry.

Physical and Ocular Characteristics	3-5 years		6-10 years		11-15 years		16-18 years		Total	Statistic Value	pValue	Test name
	Male	Female	Male	Female	Male	Female	Male	Female				
Number of children	94	56	382	247	503	547	171	174	2174			
Myopia grade, n (%)												
Mild	23 (24.5) 95% CI= (16.4%, 34.6%)	13 (23.2) 95% CI= (13.4%, 36.7%)	126 (33.0) 95% CI= (28.3%, 38.0%)	73 (29.6) 95% CI= (24.0%, 35.7%)	224 (44.5) 95% CI= (40.1%, 49.0%)	256 (46.8) 95% CI= (42.6%, 51.1%)	83 (48.5) 95% CI= (40.9%, 56.3%)	72 (41.4) 95% CI= (34.1%, 49.1%)	870 (40.0) 95% CI= (38.0%, 42.1%)	139.6	<0.001	Chisq
Moderate	16 (17.0) 95% CI= (10.3%, 26.5%)	10 (17.9) 95% CI= (9.34%, 30.8%)	91 (23.8) 95% CI= (19.7%, 28.5%)	81 (32.8) 95% CI= (27.1%, 39.1%)	162 (32.2) 95% CI= (28.2%, 36.5%)	176 (32.2) 95% CI= (28.3%, 36.3%)	50 (29.2) 95% CI= (22.7%, 36.8%)	47 (27.0) 95% CI= (20.7%, 34.4%)	633 (29.1) 95% CI= (27.2%, 31.1%)			
Severe	55 (58.5) 95% CI= (47.9%, 68.4%)	33 (58.9) 95% CI= (45.0%, 71.6%)	165 (43.2) 95% CI= (38.2%, 48.3%)	93 (37.7) 95% CI= (31.6%, 44.0%)	117 (23.3) 95% CI= (19.7%, 27.3%)	115 (21.0) 95% CI= (17.7%, 24.7%)	38 (22.2) 95% CI= (16.4%, 29.3%)	55 (31.6) 95% CI= (24.9%, 39.1%)	671 (30.9) 95% CI= (28.9%, 32.9%)			
Vision and Refractive Error (Mean ± SD)												
BCVA (LogMAR)	0.37 ± 0.31 95% CI= (0.296, 0.436)	0.38 ± 0.29 95% CI= (0.292, 0.458)	0.20 ± 0.29 95% CI= (0.171, 0.230)	0.19 ± 0.26 95% CI= (0.158, 0.223)	0.09 ± 0.18 95% CI= (0.070, 0.101)	0.06 ± 0.18 95% CI= (0.050, 0.079)	0.06 ± 0.18 95% CI= (0.035, 0.089)	0.05 ± 0.16 95% CI= (0.031, 0.078)	0.12 ± 0.23 95% CI= (0.115, 0.135)	338.7	<0.001	Kruskal-walis
Cycloplegic Sphere (Dioptres)	-7.38 ± 5.09 95% CI= (-8.42, -6.33)	-7.35 ± 4.69 95% CI= (-8.62, -6.08)	-5.43 ± 4.05 95% CI= (-5.84, -5.02)	-5.06 ± 3.72 95% CI= (-5.53, -4.59)	-4.21 ± 3.73 95% CI= (-4.54, -3.88)	-3.85 ± 3.51 95% CI= (-4.15, -3.56)	-3.99 ± 3.33 95% CI= (-4.50, -3.48)	-4.49 ± 3.31 95% CI= (-4.98, -3.99)	-4.65 ± 3.88 95% CI= (-4.82, -4.49)	113.6	<0.001	Kruskal-walis
Cycloplegic Cylinder (Dioptres)	-1.90 ± 0.98 95% CI= (-2.12, -1.68)	-2.14 ± 1.23 95% CI= (-2.51, -1.78)	-1.57 ± 1.07 95% CI= (-1.69, -1.45)	-1.87 ± 1.06 95% CI= (-2.02, -1.72)	-1.38 ± 1.01 95% CI= (-1.48, -1.27)	-1.48 ± 1.19 95% CI= (-1.59, -1.36)	-1.43 ± 0.95 95% CI= (-1.61, -1.25)	-1.43 ± 1.06 95% CI= (-1.62, -1.24)	-1.55 ± 1.10 95% CI= (-1.61, -1.50)	76.6	<0.001	Kruskal-walis
Cycloplegic SE (Dioptres)	-8.09 ± 5.22 95% CI= (-9.15, -7.02)	-8.12 ± 5.04 95% CI= (-9.47, -6.77)	-6.05 ± 4.24 95% CI= (-6.47, -5.62)	-5.78 ± 3.99 95% CI= (-6.28, -5.28)	-4.66 ± 3.94 95% CI= (-5.00, -4.31)	-4.36 ± 3.73 95% CI= (-4.67, -4.05)	-4.34 ± 3.59 95% CI= (-4.88, -3.80)	-4.96 ± 3.57 95% CI= (-5.49, -4.43)	-5.19 ± 4.11 95% CI= (-5.36, -5.02)	123.1	<0.001	Kruskal-walis
Ocular Biometric Parameters (Mean ± SD)												
Axial Length (mm)	25.49 ± 1.97 95% CI= (25.1, 25.9)	24.84 ± 1.93 95% CI= (24.3, 25.4)	25.26 ± 1.64 95% CI= (25.1, 25.4)	24.72 ± 1.52 95% CI= (24.5, 24.9)	25.13 ± 1.51 95% CI= (25.0, 25.3)	24.62 ± 1.65 95% CI= (24.5, 24.8)	25.23 ± 1.66 95% CI= (25.0, 25.5)	24.78 ± 1.49 95% CI= (24.6, 25.0)	24.97 ± 1.64 95% CI= (24.9, 25.0)	79.8	<0.001	Kruskal-walis
AL/CR Ratio	3.34 ± 0.24 95% CI= (3.29, 3.39)	3.32 ± 0.26 95% CI= (3.25, 3.39)	3.30 ± 0.22 95% CI= (3.28, 3.33)	3.28 ± 0.21 95% CI= (3.26, 3.31)	3.29 ± 0.19 95% CI= (3.27, 3.31)	3.26 ± 0.21 95% CI= (3.24, 3.28)	3.25 ± 0.21 95% CI= (3.22, 3.29)	3.27 ± 0.20 95% CI= (3.24, 3.30)	3.28 ± 0.21 95% CI= (3.27, 3.29)	32.9	<0.001	Kruskal-walis
Anterior Chamber Depth (mm)	3.56 ± 0.29 95% CI= (3.50, 3.62)	3.49 ± 0.35 95% CI= (3.39, 3.59)	3.68 ± 0.27 95% CI= (3.65, 3.71)	3.62 ± 0.25 95% CI= (3.59, 3.66)	3.76 ± 0.27 95% CI= (3.74, 3.78)	3.73 ± 0.26 95% CI= (3.70, 3.75)	3.76 ± 0.33 95% CI= (3.71, 3.81)	3.68 ± 0.26 95% CI= (3.64, 3.72)	3.70 ± 0.28 95% CI= (3.69, 3.71)	99.4	<0.001	Kruskal-walis
Lens Thickness (mm)	3.50 ± 0.19 95% CI= (3.45, 3.55)	3.49 ± 0.14 95% CI= (3.43, 3.54)	3.45 ± 0.24 95% CI= (3.42, 3.48)	3.44 ± 0.22 95% CI= (3.40, 3.47)	3.43 ± 0.21 95% CI= (3.40, 3.45)	3.45 ± 0.22 95% CI= (3.43, 3.47)	3.42 ± 0.28 95% CI= (3.37, 3.47)	3.42 ± 0.21 95% CI= (3.39, 3.46)	3.44 ± 0.23 95% CI= (3.43, 3.45)	13.8	0.054	Kruskal-walis
Central Corneal Thickness (Microns)	516.03 ± 42.64 95% CI= (501, 531)	503.07 ± 42.33 95% CI= (479, 528)	525.80 ± 30.81 95% CI= (521, 530)	523.36 ± 33.44 95% CI= (517, 529)	529.72 ± 34.18 95% CI= (526, 534)	536.70 ± 38.88 95% CI= (532, 541)	529.06 ± 33.10 95% CI= (521, 537)	525.40 ± 30.61 95% CI= (519, 532)	528.85 ± 35.27 95% CI= (527, 531)	22.6	0.002	Kruskal-walis
K1 (Dioptres)	43.45 ± 1.95 95% CI= (43.1, 43.9)	44.23 ± 1.57 95% CI= (43.8, 44.7)	43.53 ± 1.81 95% CI= (43.3, 43.7)	44.10 ± 1.60 95% CI= (43.9, 44.3)	43.73 ± 1.88 95% CI= (43.6, 43.9)	44.15 ± 1.63 95% CI= (44.0, 44.3)	43.11 ± 1.73 95% CI= (42.8, 43.4)	43.97 ± 1.66 95% CI= (43.7, 44.2)	43.81 ± 1.77 95% CI= (43.7, 43.9)	92.6	<0.001	Kruskal-walis
K2 (Dioptres)	45.05 ± 2.40 95% CI= (44.6, 45.5)	46.01 ± 1.78 95% CI= (45.5, 46.5)	44.80 ± 1.85 95% CI= (44.6, 45.0)	45.49 ± 1.77 95% CI= (45.3, 45.7)	44.75 ± 1.91 95% CI= (44.6, 44.9)	45.28 ± 1.69 95% CI= (45.1, 45.4)	44.15 ± 1.78 95% CI= (43.9, 44.4)	45.16 ± 1.90 95% CI= (44.9, 45.4)	45.00 ± 1.88 95% CI= (44.9, 45.1)	117.0	<0.001	Kruskal-walis
Average K (Dioptres)	44.25 ± 2.07 95% CI= (43.8, 44.7)	45.12 ± 1.53 95% CI= (44.7, 45.5)	44.16 ± 1.70 95% CI= (44.0, 44.3)	44.79 ± 1.56 95% CI= (44.6, 45.0)	44.24 ± 1.78 95% CI= (44.1, 44.4)	44.71 ± 1.53 95% CI= (44.6, 44.8)	43.63 ± 1.71 95% CI= (43.4, 43.9)	44.57 ± 1.69 95% CI= (44.3, 44.8)	44.41 ± 1.71 95% CI= (44.3, 44.5)	115.8	<0.001	Kruskal-walis

Table 3 shows the distribution of the demographics, vision, refractive error, and ocular biometry among males and females. The males in the study population were slightly younger and engaged in greater hours of outdoor activities and fewer hours of indoor activities than females. The mean SE in the males was slightly greater than in females (-5.35±4.24 D vs -5.01±3.96 D). The AL, AL/CR ratio, and ACD were higher in males, while the K were higher in females. The LT and CCT were similar between the two genders.

**Table 3:** Distribution of demographic, clinical, and ocular biometric characteristics among the males and females CI - Confidence Interval, BCVA – Best Corrected Visual Acuity, SD – Standard Deviation, BMI - Body Mass Index, SE – Spherical Equivalent, AL/CR ratio – Axial Length to Corneal Curvature ratio, K – Keratometry

Parameters	Male	Female	Total	Test Statistic	p-value	Test name
Myopia Category						
Mild	456 (39.7%) 95% CI= (36.8%, 42.6%)	414 (40.4%) 95% CI= (37.4%, 43.5%)	870 (40.0%) 95% CI= (38.0%, 42.1%)	4.08	0.13	Chi-sq
Moderate	319 (27.7%) 95% CI= (25.2%, 30.4%)	314 (30.7%) 95% CI= (27.9%, 33.6%)	633 (29.1%) 95% CI= (27.2%, 31.1%)			
Severe	375 (32.6%) 95% CI= (29.9%, 35.4%)	296 (28.9%) 95% CI= (26.2%, 31.8%)	671 (30.9%) 95% CI= (28.9%, 32.9%)			
Personal Characteristics (Mean ± SD)						
Age (years)	11.29 ± 3.87 95% CI= (11.1, 11.5)	11.96 ± 3.53 95% CI= (11.7, 12.2)	11.61 ± 3.73 95% CI= (11.4, 11.8)	642607	<0.001	Wilcoxon
Height (cms)	1.35 ± 0.26 95% CI= (1.33, 1.37)	1.38 ± 0.20 95% CI= (1.37, 1.40)	1.37 ± 0.23 95% CI= (1.35, 1.38)	230643	0.076	Wilcoxon
Weight (kg)	37.04 ± 15.93 95% CI= (35.9, 38.2)	36.55 ± 12.51 95% CI= (35.6, 37.5)	36.81 ± 14.41 95% CI= (36.0, 37.6)	224104.5	0.400	Wilcoxon
BMI	19.36 ± 5.38 95% CI= (19.0, 19.8)	18.77 ± 4.86 95% CI= (18.4, 19.2)	19.08 ± 5.15 95% CI= (18.8, 19.4)	200792	0.200	Wilcoxon
Outdoor and near activities (Mean ± SD)						
Outdoor Activities (hours/day)	1.74 ± 1.83 95% CI= (1.61, 1.88)	1.07 ± 1.60 95% CI= (0.948, 1.20)	1.43 ± 1.76 95% CI= (1.33, 1.52)	162713.5	<0.001	Wilcoxon
Near Activities (hours/day)	5.71 ± 4.10 95% CI= (5.40, 6.02)	6.51 ± 4.16 95% CI= (6.16, 6.87)	6.06 ± 4.14 95% CI= (5.83, 6.30)	198242	<0.001	Wilcoxon
Vision and Refractive Error (Mean ± SD)						
BCVA (LogMAR)	0.14 ± 0.25 95% CI= (0.125, 0.154)	0.11 ± 0.22 95% CI= (0.095, 0.122)	0.12 ± 0.23 95% CI= (0.115, 0.135)	526138	0.003	Wilcoxon
Cycloplegic Sphere (Diopters)	-4.84 ± 4.02 95% CI= (-5.07, -4.61)	-4.44 ± 3.69 95% CI= (-4.67, -4.21)	-4.65 ± 3.88 95% CI= (-4.82, -4.49)	609441	0.035	Wilcoxon
Cycloplegic Cylinder (Diopters)	-1.50 ± 1.03 95% CI= (-1.57, -1.43)	-1.61 ± 1.16 95% CI= (-1.69, -1.53)	-1.55 ± 1.10 95% CI= (-1.61, -1.50)	315416	0.3	Wilcoxon
Cycloplegic SE (Diopters)	-5.35 ± 4.24 95% CI= (-5.60, -5.11)	-5.01 ± 3.96 95% CI= (-5.25, -4.77)	-5.19 ± 4.11 95% CI= (-5.36, -5.02)	611159.5	0.13	Wilcoxon
Ocular Biometric Parameters (Mean ± SD)						
Axial length (mm)	25.22 ± 1.62 95% CI= (25.1, 25.3)	24.68 ± 1.61 95% CI= (24.6, 24.8)	24.97 ± 1.64 95% CI= (24.9, 25.0)	465259	<0.001	Wilcoxon
AL/CR Ratio	3.29 ± 0.21 95% CI= (3.28, 3.31)	3.27 ± 0.21 95% CI= (3.26, 3.28)	3.28 ± 0.21 95% CI= (3.27, 3.29)	521683	<0.001	Wilcoxon
Anterior Chamber Depth (mm)	3.72 ± 0.29 95% CI= (3.70, 3.73)	3.68 ± 0.27 95% CI= (3.66, 3.70)	3.70 ± 0.28 95% CI= (3.69, 3.71)	515445.5	0.005	Wilcoxon
Lens Thickness (mm)	3.44 ± 0.24 95% CI= (3.42, 3.45)	3.44 ± 0.22 95% CI= (3.43, 3.46)	3.44 ± 0.23 95% CI= (3.43, 3.45)	269248	0.8	Wilcoxon
Central Corneal Thickness (Microns)	527.49 ± 33.65 95% CI= (525, 530)	530.35 ± 36.95 95% CI= (527, 534)	528.85 ± 35.27 95% CI= (527, 531)	137736	0.2	Wilcoxon
K1 (Diopters)	43.55 ± 1.85 95% CI= (43.4, 43.7)	44.11 ± 1.62 95% CI= (44.0, 44.2)	43.81 ± 1.77 95% CI= (43.7, 43.9)	691590.5	<0.001	Wilcoxon
K2 (Diopters)	44.70 ± 1.93 95% CI= (44.6, 44.8)	45.35 ± 1.76 95% CI= (45.2, 45.5)	45.00 ± 1.88 95% CI= (44.9, 45.1)	702672.5	<0.001	Wilcoxon
Average K (Diopters)	44.12 ± 1.78 95% CI= (44.0, 44.2)	44.73 ± 1.57 95% CI= (44.6, 44.8)	44.41 ± 1.71 95% CI= (44.3, 44.5)	706540.5	<0.001	Wilcoxon

Table 4 displays physical characteristics, vision, and ocular biometry across mild, moderate, and severe myopia categories. The average age, height, weight, and BMI decreased with increasing severity of myopia (p<0.001).

**Table 4:** Physical characteristics, vision, and ocular biometry across mild, moderate, and severe myopia categories CI: Confidence Interval, BMI: Body Mass Index, SD: Standard Deviation, BCVA: Best Corrected Visual Acuity, SE: Spherical Equivalent, AL/CR ratio: Axial Length to Corneal curvature ratio, K: Keratometry.

	Mild Myopia	Moderate Myopia	Severe Myopia	Total	Test Statistic	p-value	Test name
Physical Characteristic (Mean ± SD)							
Age (years)	12.29 ± 3.37 95% CI= (12.1, 12.5) (n=870)	12.03 ± 3.42 95% CI= (11.8, 12.3) (n=633)	10.32 ± 4.11 95% CI= (10.0, 10.6) (n=671)	11.61 ± 3.73 95% CI= (11.4, 11.8) (n=2174)	99.23	<0.001	Kruskal Wallis
Height (meters)	1.40 ± 0.23 95% CI= (1.38, 1.41) (n=608)	1.38 ± 0.21 95% CI= (1.36, 1.40) (n=394)	1.29 ± 0.24 95% CI= (1.27, 1.32) (n=322)	1.37 ± 0.23 95% CI= (1.35, 1.38) (n=1324)	47.31	<0.001	Kruskal Wallis
Weight (kg)	39.81 ± 13.85 95% CI= (38.7, 40.9) (n=610)	37.56 ± 14.64 95% CI= (36.1, 39.0) (n=394)	30.16 ± 12.98 95% CI= (28.7, 31.6) (n=320)	36.81 ± 14.41 95% CI= (36.0, 37.6) (n=1324)	107.68	<0.001	Kruskal Wallis
BMI	19.90 ± 5.23 95% CI= (19.5, 20.3) (n=591)	19.33 ± 5.21 95% CI= (18.8, 19.8) (n=391)	17.22 ± 4.42 95% CI= (16.7, 17.7) (n=314)	19.08 ± 5.15 95% CI= (18.8, 19.4) (n=1296)	63.40	<0.001	Kruskal Wallis
Outdoor and Indoor Activities (Mean ± SD)							
Outdoor Activities (hours/day)	1.31 ± 1.48 95% CI= (1.19, 1.43) (n=605)	1.42 ± 1.69 95% CI= (1.25, 1.59) (n=385)	1.64 ± 2.21 95% CI= (1.40, 1.87) (n=340)	1.43 ± 1.76 95% CI= (1.33, 1.52) (n=1330)	0.51	0.8	Kruskal Wallis
Near Activities (hours/day)	6.02 ± 3.91 95% CI= (5.69, 6.35) (n=539)	6.37 ± 4.29 95% CI= (5.93, 6.80) (n=376)	5.74 ± 4.34 95% CI= (5.23, 6.24) (n=287)	6.06 ± 4.14 95% CI= (5.83, 6.30) (n=1202)	4.26	0.12	Kruskal Wallis
Vision and Refractive Errors (Mean ± SD)							
BCVA (LogMAR)	0.03 ± 0.09 95% CI= (0.027, 0.040) (n=859)	0.09 ± 0.17 95% CI= (0.074, 0.101) (n=628)	0.28 ± 0.32 95% CI= (0.259, 0.309) (n=639)	0.12 ± 0.23 95% CI= (0.115, 0.135) (n=2126)	516.76	<0.001	Kruskal Wallis
Cyclo Sphere (Dioptres)	-1.68 ± 0.72 95% CI= (-1.73, -1.63) (n=852)	-3.78 ± 0.96 95% CI= (-3.86, -3.71) (n=633)	-9.25 ± 3.70 95% CI= (-9.53, -8.97) (n=671)	-4.65 ± 3.88 95% CI= (-4.82, -4.49) (n=2156)	1,813.17	<0.001	Kruskal Wallis
Cyclo Cylinder (Dioptres)	-0.99 ± 0.77 95% CI= (-1.06, -0.923) (n=494)	-1.42 ± 1.04 95% CI= (-1.51, -1.32) (n=508)	-2.12 ± 1.10 95% CI= (-2.21, -2.04) (n=612)	-1.55 ± 1.10 95% CI= (-1.61, -1.50) (n=1614)	366.34	<0.001	Kruskal Wallis
Cyclo SE (Dioptres)	-1.93 ± 0.71 95% CI= (-1.97, -1.88) (n=870)	-4.35 ± 0.84 95% CI= (-4.41, -4.28) (n=633)	-10.22 ± 3.70 95% CI= (-10.5, -9.94) (n=671)	-5.19 ± 4.11 95% CI= (-5.36, -5.02) (n=2174)	1,917.26	<0.001	Kruskal Wallis
Ocular Biometry Parameters							
Axial length (mm)	23.97 ± 0.92 95% CI= (23.9, 24.0) (n=870)	24.73 ± 0.94 95% CI= (24.7, 24.8) (n=632)	26.48 ± 1.77 95% CI= (26.3, 26.6) (n=671)	24.97 ± 1.64 95% CI= (24.9, 25.0) (n=2173)	989.40	<0.001	Kruskal Wallis
AL/CR Ratio	3.15 ± 0.14 95% CI= (3.14, 3.16) (n=849)	3.25 ± 0.12 95% CI= (3.24, 3.26) (n=626)	3.48 ± 0.21 95% CI= (3.47, 3.50) (n=664)	3.28 ± 0.21 95% CI= (3.27, 3.29) (n=2139)	1,082.27	<0.001	Kruskal Wallis
Anterior Chamber Depth (mm)	3.74 ± 0.26 95% CI= (3.72, 3.75) (n=845)	3.72 ± 0.27 95% CI= (3.70, 3.74) (n=614)	3.63 ± 0.30 95% CI= (3.61, 3.66) (n=652)	3.70 ± 0.28 95% CI= (3.69, 3.71) (n=2111)	56.00	<0.001	Kruskal Wallis
Lens Thickness (mm)	3.46 ± 0.24 95% CI= (3.44, 3.48) (n=586)	3.41 ± 0.22 95% CI= (3.39, 3.43) (n=422)	3.44 ± 0.21 95% CI= (3.42, 3.46) (n=465)	3.44 ± 0.23 95% CI= (3.43, 3.45) (n=1473)	12.86	0.002	Kruskal Wallis
Central Corneal Thickness (Microns)	532.09 ± 32.15 95% CI= (528, 536) (n=309)	530.89 ± 36.56 95% CI= (527, 535) (n=351)	524.18 ± 36.11 95% CI= (520, 528) (n=369)	528.85 ± 35.27 95% CI= (527, 531) (n=1029)	9.24	0.01	Kruskal Wallis
K1 (Diopters)	44.02 ± 1.80 95% CI= (43.9, 44.1) (n=849)	43.75 ± 1.75 95% CI= (43.6, 43.9) (n=626)	43.61 ± 1.72 95% CI= (43.5, 43.7) (n=665)	43.81 ± 1.77 95% CI= (43.7, 43.9) (n=2140)	19.81	<0.001	Kruskal Wallis
K2 (Diopters)	44.81 ± 1.86 95% CI= (44.7, 44.9) (n=848)	44.95 ± 1.82 95% CI= (44.8, 45.1) (n=626)	45.30 ± 1.93 95% CI= (45.2, 45.4) (n=665)	45.00 ± 1.88 95% CI= (44.9, 45.1) (n=2139)	29.17	<0.001	Kruskal Wallis
Average K (Diopters)	44.42 ± 1.71 95% CI= (44.3, 44.5) (n=849)	44.35 ± 1.68 95% CI= (44.2, 44.5) (n=626)	44.45 ± 1.73 95% CI= (44.3, 44.6) (n=664)	44.41 ± 1.71 95% CI= (44.3, 44.5) (n=2139)	1.95	0.4	Kruskal Wallis

The average duration of near activities did not differ significantly between the three grades of myopia. However, the average duration of outdoor activities increased with the grade of myopia. The BCVA worsened with the severity of myopia. The average cylinder increased with the severity of myopia, with the flatter K (K1) becoming flatter and the steeper K (K2) becoming steeper. The AL and AL/CR ratio increased with myopia's severity, while the average K remained the same. The ACD and CCT reduced with the severity of myopia (p<0.001 and p=0.006, respectively). Although LT also changed significantly across the groups, the change was not unidirectional. It was lowest in the moderate myopia group (3.41 ± 0.22 mm). The average LT in mild and severe myopic eyes was 3.46 ± 0.24 mm and 3.44 ± 0.21 mm, respectively.

**Figure 3:**
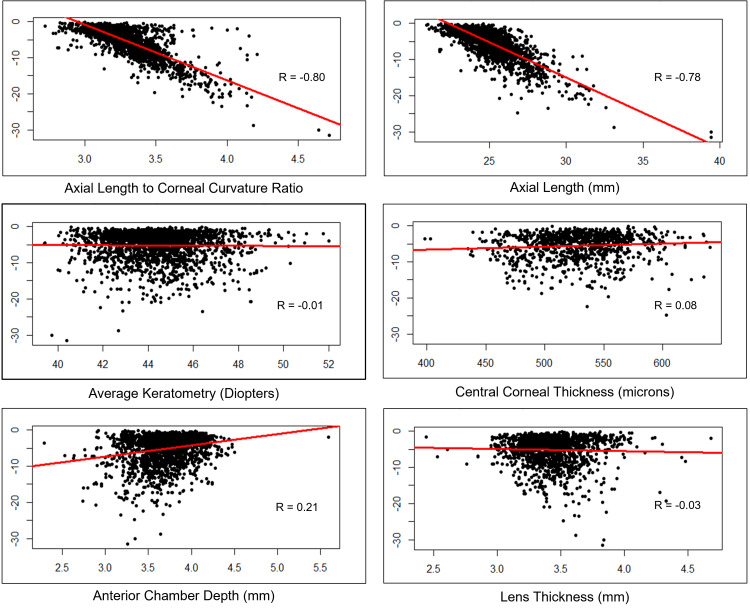
Correlation of the spherical equivalent with the various ocular biometric parameters The value on the Y-axis represents the spherical equivalent in diopters. R = coefficient of correlation

Figure 3 shows Pearson’s correlation between the severity of myopia (measured by SE) and various ocular biometry parameters, along with corresponding scatter plots. Axial length and AL/CR ratio emerged as critical predictors of SE in myopic children. Both increased as the severity of myopia increased (R=0.80 and R=0.78 for AL and AL/CR ratio, respectively). Pearson’s correlation coefficient between SE and ACD was 0.21, while the correlation coefficients were negligibly small for other ocular biometric parameters. 

Multivariable analysis 

The results of the final multivariable model are presented in Table 5. Axial length showed a strong association with spherical equivalent, with longer eyes exhibiting more myopia (estimate = -2.53, p < 0.001). Average keratometry also demonstrated a clear relationship, where steeper corneas were linked to greater myopic refractive error (estimate = -1.01, p < 0.001). Both anterior chamber depth and lens thickness were significantly associated with myopia. Deeper anterior chambers were related to slightly lesser myopia (estimate = 2.73, p < 0.001), whereas increasing lens thickness was associated with greater myopia (estimate = -1.63, p = 0.001). Central corneal thickness showed no meaningful independent association (p = 0.743). Among the personal characteristics, height displayed a modest positive association, with taller children showing marginally less myopia (estimate = 3.70, p < 0.001). BMI had a borderline p-value with a negligible effect size (estimate = 0.05, p = 0.071). Parental myopia and several other covariates were not significant after adjusting for the other variables.

**Table 5:** Results of multivariable mix effect regression analysis (outcome=cycloplegic spherical equivalent), with children as a random factor CI: Confidence Interval, ACD: Anterior Chamber Depth, BMI: Body Mass Index, CCT: Central Corneal Thickness Random Effects: Children (Intercept) 1.71 (SD=1.31) Residual 0.45 (SD=0.67) Number of observations = 372 p-value < 0.001 (Restricted Likelihood Ratio Test) Adjusted ICC: 0.791 Unadjusted ICC: 0.123. Model Fit Statistics:  Conditional R-square = 0.968 Marginal R-square = 0.845.

	Estimate	95% CI Lower Limit		95% CI Upper Limit	Value of Test Statistic	pValue
(Intercept)	92.17	83.10		101.46	19.493	0.000
Height	3.70	2.60		4.81	6.452	0.000
ACD	2.73	1.87		3.59	6.133	0.000
Axial length	-2.53	-2.64		-2.41	-43.868	0.000
Lens Thickness	-1.63	-2.56		-0.72	-3.456	0.001
Average K	-1.01	-1.13		-0.89	-16.779	< 0.001
Myopia Father	-0.28	-0.82		0.27	-0.984	0.327
Myopia Mother	0.24	-0.29		0.77	0.881	0.380
BMI	0.05	0.00		0.10	1.813	0.071
CCT	0.00	-0.01		0.00	-0.328	0.743

## Discussion

The ocular biometric parameters are crucial in understanding, diagnosing, and managing myopia. They provide insights into the anatomical and optical changes that happen with the progression of myopia. Axial length reflects true structural elongation, while SE may be affected by accommodation and lens changes as well. It is the key outcome in myopia control trials and can also identify patients at risk of rapid progression [39,40]. The corneal power influences baseline refractive status and interacts with axial length to determine refractive error. Changes in corneal curvature, lens thickness, and AC depth may mask an underlying axial progression if SE alone is taken as a measure of myopia progression. In the current study, we analysed the changes in the ocular biometric parameters in children with myopia and correlated them with the degree of myopia. Multivariable analysis revealed that AL, CR, ACD, and LT were independently associated with the severity of myopia. Among the physical characteristics, shorter child height was significantly associated with severe myopia.

The AL/CR ratio had a positive linear relationship with the severity of myopia in the study patients in the univariable analysis. This concurs with the results of prior studies in young adults [33,41] and children [15,23-28,32,34,37]. Grosvenor and Scott suggested that the AL/CR ratio predicts the refractive error better than AL alone [42]. An AL/CR ratio of > 3 suggests myopia [32]. Minor AL/CR ratio changes can lead to large changes in the refractive error [31,43]. However, in the current study, the AL/CR ratio did not retain independent significance in the multivariable model, since both AL and CR were independently and concordantly associated with SE. Harrington and O’Dwyer studied the ocular biometric parameters in 1626 Irish school children in two age groups: six to seven years and 12 to 13 years [24]. The children suffered from all refractive errors, ranging from +8 to -10D. Their study found a similar relationship between both AL and AL/CR ratios with SE. Liu et al. reported the biometry in 902 Chinese children aged six to 14 years (mean 10.03 ± 2.47 years), including refractive error ranging from mild hyperopia to mild myopia (+3.0 to -3.0D) [25]. An increase of 1mm of AL was associated with a 0.69D increase in myopia in their patients. A similar correlation between SE and AL was observed by Hashemi et al. in a study of 4938 Iranian children aged six to 12 years, including all types of refractive errors [28]. We included only myopic children to correlate the changes in ocular biometry with the severity of myopia. A recent study of ocular biometry of 3728 myopic children from South India also found that the AL/CR ratio could explain 71% of the total variance in SE [37]. As evident from the literature discussed above and the current study, AL is the most crucial determinant of the severity of myopia. 

The eye's refractive status is also determined by CR and LT. The CR has been shown to correlate negatively with the AL, wherein the cornea flattens to compensate for the increase in AL [15]. This correlation exists over a range of AL and may not hold for extremely long or short eyes. It might also be different in juvenile myopia. In the current study, the average K remained similar between the three grades of myopia. Similar observations were made by Saw et al. [44] in the SCORM (Singapore Cohort Study of the Risk Factors for Myopia) study and by Li et al. [32].The results of the longitudinal SCORM study showed that while the AL increased by an average of 0.89mm, the corneal radii remained stable with a 0.01mm change, concluding that CR did not influence myopia progression. The Correction of Myopia Evaluation Trial (COMET) cohort was followed up for 14 years, and it was found that the CR flattened slightly (from 44.11±1.40 to 43.98±1.44) but significantly (p<0.0001) [45]. The flatter meridian became flatter while the steeper meridian remained unchanged. Interestingly, the flat K was similar in the three groups of Li et al.'s study, while the steeper K increased in the severe myopia group [32]. In the current study, the flatter K became flatter, and the steeper K became steeper in the severe myopia group compared to the mild and moderate myopia groups. This led to a more significant astigmatism in the severe myopia group, keeping the average K unchanged. As this is a cross-sectional study, the results of the current investigation cannot be compared directly with those of the COMET or SCORM studies. However, ethnicity playing a role in these differences may be a possibility. 

Studies have shown that ACD increases with myopia while the LT or lens power reduces [15,24,25,32,33]. These findings support the mechanical tension theory proposed by Mutti et al., in which the crystalline lens or ciliary body restricts equatorial ocular expansion during accommodation and causes axial elongation instead [46]. This leads to an increase in corneal curvature, deepening of the ACD, an increase in the vitreous chamber depth and thinning of the crystalline lens. Li et al., who compared the different grades of myopia in a pediatric population, found no difference in the ACD between the three groups [32]. However, they found that the AL positively correlated with the ACD in the highly myopic eyes. In the current study, we found a negative association between ACD and the severity of myopia, meaning that ACD was lower in the severe myopia group. There is a possibility that LT did not decline in proportion to the AL in the current study patients, leading to reduced ACD despite increased vitreous chamber depth. While the LT reduced from mild to moderate myopia, supporting the mechanical tension theory, it remained higher in severely myopic patients. In a large study (n=11,656), Shih et al. reported that LT reduced from ages seven to 11 years and increased after that despite a gradual increase in average myopia [47]. It must be noted that the age of the patients in the severe myopia category in the current study was lower than that of the mild and moderate groups in our study. Therefore, whether this relationship between ACD and LT denotes a unique pattern of eye growth in young children with high myopia is a matter for future longitudinal studies. 

In the current study, we observed a negative association between CCT and the severity of myopia in the univariable analysis and no association in the multivariable model. The association of CCT with the degree of myopia is unclear in the literature. Some studies show a negative association wherein the CCT is lower in higher grades of myopia [33,48]. Other studies, mainly in adults, have shown no correlation between CCT and the degree of myopia [15,49]. The importance of CCT in myopic eyes was highlighted by Zhou et al., who found that a thinner CCT, not ACD or K, was a risk factor for AL elongation and myopia progression [50]. However, they did not consider the magnitude of the baseline myopia as one of the factors in their study. As faster progression and thinner central corneal thickness are both associated with severe myopia, this relationship is likely subject to confounding.

The differences in height, weight, and BMI among the three grades of myopia in the current study reflect the difference in the average age of the children between the three groups. Since myopia increases with age, most prior studies have found a positive correlation between height, BMI, and severity of myopia [51]. In the current study, the children in the severe grade of myopia were younger (hence shorter height). We selected the patients in the current study from a clinic-based population and included only those who had undergone biometric analysis. This might have led to the skewing of the severity of myopia towards a younger age. 

We observed that the children with severe myopia tended to spend more time outdoors and less time in indoor activities when compared to those with mild to moderate myopia. Although the p-value indicates statistical significance (p=0.024), the absolute difference in the average duration of outdoor activities between the groups, varying from 10 to 20 minutes, was not clinically relevant. Nonetheless, this difference could be due to the influence of counselling regarding lifestyle modifications by the eye care professionals and the severe myopes being more receptive to their advice. The differences in the ocular biometric parameters and gender in the current study are similar to those in another hospital-based study from Southern India [37]. The AL/CR ratio was the same across all ages. Using ANOVA, the authors correlated the AL, AL/CR, and CR with the severity of myopia. They found that the first two increased with the degree of myopia, whereas the latter reduced. 

The major limitation of the current study is its retrospective design. Longitudinal follow-up data of the same patients were not collected, which could show the temporal changes in ocular parameters with the shifts in SE, thereby providing deeper insight into myopia development and progression. We did not exclude nor analyse strabismus and amblyopia as factors affecting BCVA. Thus, the association between the severity of myopia and BCVA must be interpreted with caution. We did not consider whether a patient was using any treatment to retard the progression of myopia, which could have influenced axial length. The exposure measures, like indoor and outdoor activities and parental myopia, were based on the history given by the parents. No validated instruments were used to measure the indoor or outdoor activities. Nonetheless, our study has several key strengths that contribute to the reliability and validity of our findings. Firstly, being multicentric, it reflects data from a diverse part of the country. An optical biometer was used at all the participating institutes. The optical biometer is an easy, non-contact procedure that provides all biometric parameters together compared to ultrasound-based biometers. It has been shown that the AL measurements may vary between different optical biometers [52]. However, IOL master 700 and 500 have shown excellent agreement in biometric measurements in emmetropic adolescents, adult cataract patients, and myopes without posterior staphyloma [53-56]. In patients with posterior staphyloma, AL measured by IOL master 700 can be overestimated compared to IOL master 500. The IOL master 500 was used in just 131 subjects (12%) for the acquisition of biometry data in this study. Thus, we do not consider the inclusion of IOL master 500 and 700-generated data to be a major limitation, particularly because this was a cross-sectional study with no longitudinal measurements in the same patients, and the two devices have demonstrated excellent agreement. Finally, the sample size is derived from a clinic-based population, and the participants may not represent the general myopic population. However, the main aim of the current study was to compare ocular biometric parameters across mild, moderate, and severe myopia. By performing multivariable analyses and adjusting for potential confounders such as age and gender, we believe that our results provide robust and internally valid associations within this clinic-based cohort.

## Conclusions

To conclude, the study presents the biometric profile of a large sample of myopic children and correlates it with the severity of myopia. The ocular biometric parameters we examined were closely interconnected with the SE. Among the ocular biometric characteristics, AL was the most important determinant of SE in myopic children. We found that the LT and cylindrical diopter increased while the ACD decreased with the severity of myopia. It is crucial to comprehend the changes in these parameters as AL and SE increase across different degrees of myopia. This understanding is essential for interpreting ocular biometric data and establishing their relationship with myopia. Future studies including longitudinal follow-up to confirm the progression patterns observed would be valuable.
